# Supplementary material for: Genomic Characterization of Rare Primary Cardiac Sarcoma Entities
Source: Diagnostics (Basel). 2023 Jan 6;13(2):214. doi: 10.3390/diagnostics13020214 (PMC9858520; doi:10.3390/diagnostics13020214)
Supplement: Supplementary file 1 [file diagnostics-13-00214-s001.zip › Supplementary Table S1.pdf]

Supplementary Table S1. Differentially expressed genes (*q*-value<0.05) between the leiomyosarcoma sample (CS11) and the group comprising cardiac angiosarcomas (CS6 and CS7) and intimal sarcomas (CS1, CS2, CS3, CS4 and CS5) (logFC = log2 fold change; logCPM = log2 counts per million; FDR = false discovery rate).

GENE SYMBOL	logFC	logCPM	p-value	FDR	GENE ID
NKAIN2	9.63	3.94	1.54E-21	4.31E-17	ENSG00000188580
STPG2	6.81	3.96	3.37E-19	4.71E-15	ENSG00000163116
TSBP1	9.52	1.16	5.18E-13	4.83E-09	ENSG00000204296
CCDC162P	5.64	4.23	4.32E-11	3.02E-07	ENSG00000203799
GJB7	7.73	1.38	1.06E-10	5.93E-07	ENSG00000164411
AC024940.1	6.86	6.00	1.40E-09	6.55E-06	ENSG00000177359
ERI1	3.39	5.54	1.95E-09	6.99E-06	ENSG00000104626
AC068587.4	5.99	2.00	2.00E-09	6.99E-06	ENSG00000283674
RPS3AP34	5.90	1.95	2.23E-08	6.93E-05	ENSG00000242607
HSPD1P9	6.98	0.39	3.25E-08	9.10E-05	ENSG00000228036
AL080317.3	4.21	3.12	6.61E-08	1.68E-04	ENSG00000272356
AC009318.1	5.46	0.98	9.78E-08	2.16E-04	ENSG00000257176
PPHLN1	3.04	6.50	1.00E-07	2.16E-04	ENSG00000134283
MFHAS1	3.22	5.19	1.48E-07	2.97E-04	ENSG00000147324
LPA	6.98	-0.49	1.92E-07	3.58E-04	ENSG00000198670
MFSD4B	3.03	5.79	3.04E-07	5.31E-04	ENSG00000173214
YAF2	3.09	6.12	3.93E-07	6.47E-04	ENSG00000015153
PTPRK	2.73	8.17	4.36E-07	6.78E-04	ENSG00000152894
RGS6	5.84	1.43	4.81E-07	7.09E-04	ENSG00000182732
AC087763.1	6.19	-0.51	5.78E-07	8.09E-04	ENSG00000270966
IGHV2-70	5.79	1.36	7.43E-07	9.91E-04	ENSG00000274576
DSPP	4.09	2.81	9.13E-07	1.16E-03	ENSG00000152591
BMP5	7.21	4.37	1.00E-06	1.22E-03	ENSG00000112175
AC109635.4	6.48	-0.49	1.71E-06	1.99E-03	ENSG00000255042
BRCA2	2.60	7.42	2.08E-06	2.33E-03	ENSG00000139618
OR7C1	5.71	0.65	2.19E-06	2.36E-03	ENSG00000127530
CPNE8	3.67	5.84	2.30E-06	2.39E-03	ENSG00000139117
AC134684.6	6.72	-0.70	2.63E-06	2.63E-03	ENSG00000284617
SOX5	3.34	5.09	3.77E-06	3.64E-03	ENSG00000134532
AC022784.1	6.12	0.35	5.20E-06	4.85E-03	ENSG00000248538
PAK5	6.67	-1.21	5.64E-06	5.10E-03	ENSG00000101349
RPL7P33	5.99	-0.39	5.99E-06	5.24E-03	ENSG00000215236
PPP1R3B	2.70	5.35	7.08E-06	6.00E-03	ENSG00000173281

OR7A5	6.60	1.19	8.88E-06	7.31E-03	ENSG00000188269
EXTL2P1	6.53	-1.26	1.08E-05	8.62E-03	ENSG00000223976
ENAM	5.52	1.25	1.21E-05	9.40E-03	ENSG00000132464
AL445487.1	6.49	-1.32	1.24E-05	9.42E-03	ENSG00000226535
SLC2A13	2.76	5.78	1.40E-05	1.03E-02	ENSG00000151229
AC009318.2	3.90	2.56	1.52E-05	1.09E-02	ENSG00000273680
AC009318.3	6.02	-1.24	1.86E-05	1.30E-02	ENSG00000274315
DUX4L27	5.63	-0.88	2.04E-05	1.39E-02	ENSG00000258794
ZCRB1	2.63	6.99	2.23E-05	1.49E-02	ENSG00000139168
TRAF3IP2-AS1	3.22	4.12	2.29E-05	1.49E-02	ENSG00000231889
ETFRF1	3.53	2.65	2.39E-05	1.52E-02	ENSG00000205707
CASC1	3.49	2.75	2.63E-05	1.64E-02	ENSG00000118307
KCNQ1OT1	3.20	5.18	2.76E-05	1.68E-02	ENSG00000269821
AC079601.1	3.92	1.10	3.17E-05	1.89E-02	ENSG00000257225
TUBE1	2.57	5.54	4.48E-05	2.61E-02	ENSG00000074935
MDM1	2.86	6.41	5.52E-05	3.16E-02	ENSG00000111554
GRIN2B	5.38	0.67	5.69E-05	3.19E-02	ENSG00000273079
SLC12A7	2.38	6.87	6.43E-05	3.53E-02	ENSG00000113504
AC022306.1	4.03	0.19	8.25E-05	4.44E-02	ENSG00000259531
IGLV3-1	4.29	4.23	8.59E-05	4.46E-02	ENSG00000211673
AC092451.2	6.00	-1.55	8.75E-05	4.46E-02	ENSG00000256079
KNOP1P1	6.00	-1.55	8.75E-05	4.46E-02	ENSG00000256852
