# Supplementary material for: Genomic Characterization of Rare Primary Cardiac Sarcoma Entities
Source: Diagnostics (Basel). 2023 Jan 6;13(2):214. doi: 10.3390/diagnostics13020214 (PMC9858520; doi:10.3390/diagnostics13020214)
Supplement: Supplementary file 1 [file diagnostics-13-00214-s001.zip › Supplementary Table S2.pdf]

Supplementary Table S2. Differentially expressed genes (*q*-value<0.05) between the osteosarcoma sample (CS9) and the group comprising cardiac angiosarcomas (CS6 and CS7) and intimal sarcomas (CS1, CS2, CS3, CS4 and CS5) (logFC = log2 fold change; logCPM = log2 counts per million; FDR = false discovery rate).

GENE SYMBOL	logFC	logCPM	p-value	FDR	GENE ID
WIF1	11.40	6.01	9.63E-29	2.70E-24	ENSG00000156076
CSMD3	8.42	4.99	1.75E-23	2.46E-19	ENSG00000164796
GRIK1	9.17	2.62	4.84E-18	4.52E-14	ENSG00000171189
COL11A2	4.98	6.20	7.42E-18	5.20E-14	ENSG00000204248
CDH10	9.25	3.06	1.06E-16	5.76E-13	ENSG00000040731
COL25A1	6.78	4.69	1.23E-16	5.76E-13	ENSG00000188517
HNRNPA1P6	6.60	3.71	1.85E-16	7.43E-13	ENSG00000229887
UNC13C	9.04	3.02	3.70E-16	1.30E-12	ENSG00000137766
CNTNAP4	9.59	3.48	4.48E-16	1.40E-12	ENSG00000152910
RGS7	10.31	1.90	8.20E-16	2.30E-12	ENSG00000182901
MUC2	8.04	4.00	5.25E-15	1.34E-11	ENSG00000198788
ZBED9	8.71	2.40	6.15E-15	1.44E-11	ENSG00000232040
CRABP1	9.26	2.73	1.86E-14	4.01E-11	ENSG00000166426
POU6F2	9.27	2.94	2.88E-14	5.77E-11	ENSG00000106536
KCNQ2	9.35	1.56	6.01E-14	1.12E-10	ENSG00000075043
DLK1	8.41	3.86	8.02E-14	1.41E-10	ENSG00000185559
RERGL	8.51	1.76	9.06E-14	1.49E-10	ENSG00000111404
OFCC1	8.73	1.74	1.53E-13	2.38E-10	ENSG00000181355
TMEM132C	8.96	2.80	2.48E-13	3.67E-10	ENSG00000181234
SEMA3A	3.99	6.20	7.32E-13	1.03E-09	ENSG00000075213
OLFM3	8.98	1.22	9.50E-13	1.24E-09	ENSG00000118733
MYH8	7.67	1.52	9.70E-13	1.24E-09	ENSG00000133020
SLC7A10	8.47	2.46	1.39E-12	1.69E-09	ENSG00000130876
KCNA1	7.49	1.97	1.80E-12	2.10E-09	ENSG00000111262
ADCY8	8.18	1.63	2.13E-12	2.39E-09	ENSG00000155897
LIX1	9.08	1.76	3.79E-12	4.05E-09	ENSG00000145721
TMEM196	9.26	0.90	3.90E-12	4.05E-09	ENSG00000173452
EYA4	6.74	5.93	5.96E-12	5.97E-09	ENSG00000112319
SNTG1	8.47	1.73	1.14E-11	1.10E-08	ENSG00000147481
GAS2	6.48	4.31	1.24E-11	1.16E-08	ENSG00000148935
SLC8A3	7.27	2.75	1.35E-11	1.22E-08	ENSG00000100678
NEFL	9.19	3.96	1.57E-11	1.37E-08	ENSG00000277586
LSAMP	7.51	2.97	1.62E-11	1.37E-08	ENSG00000185565

NKAIN3	8.42	1.12	2.61E-11	2.10E-08	ENSG00000185942
FMN2	7.76	1.47	2.62E-11	2.10E-08	ENSG00000155816
FBN3	7.91	3.84	3.37E-11	2.62E-08	ENSG00000142449
EPHA7	8.77	5.37	3.71E-11	2.81E-08	ENSG00000135333
NDST3	7.04	2.65	5.51E-11	4.07E-08	ENSG00000164100
RGS6	7.18	2.64	1.15E-10	8.28E-08	ENSG00000182732
KCTD8	8.64	0.36	1.94E-10	1.36E-07	ENSG00000183783
TNMD	7.85	2.62	2.07E-10	1.42E-07	ENSG00000000005
ISL1	8.62	0.34	2.18E-10	1.46E-07	ENSG00000016082
USH1C	7.80	3.82	2.28E-10	1.47E-07	ENSG00000006611
KANK4	7.04	3.05	2.31E-10	1.47E-07	ENSG00000132854
PROKR2	8.18	0.41	2.96E-10	1.84E-07	ENSG00000101292
MPPED1	8.49	0.23	4.73E-10	2.84E-07	ENSG00000186732
SOX5P1	8.37	0.68	4.76E-10	2.84E-07	ENSG00000254376
SYCP1	7.95	0.70	5.70E-10	3.33E-07	ENSG00000198765
ADAMTS20	6.68	3.25	1.00E-09	5.73E-07	ENSG00000173157
PCDH11Y	7.22	2.15	1.63E-09	9.13E-07	ENSG00000099715
COL26A1	6.78	1.03	1.66E-09	9.13E-07	ENSG00000160963
NRXN1	6.43	2.54	1.69E-09	9.14E-07	ENSG00000179915
CHRNG	8.12	-0.09	3.43E-09	1.81E-06	ENSG00000196811
SYT4	7.70	0.08	3.72E-09	1.93E-06	ENSG00000132872
ZNF804B	7.98	1.71	4.36E-09	2.22E-06	ENSG00000182348
CDH7	7.64	0.04	4.67E-09	2.34E-06	ENSG00000081138
TOX3	8.26	1.57	4.85E-09	2.39E-06	ENSG00000103460
NEFM	6.73	1.88	5.06E-09	2.44E-06	ENSG00000104722
FBXL21	6.96	2.28	5.28E-09	2.51E-06	ENSG00000164616
MUCL1	8.03	-0.16	5.99E-09	2.80E-06	ENSG00000172551
SHISA3	7.05	2.91	6.55E-09	3.01E-06	ENSG00000178343
UNCX	7.99	-0.21	7.30E-09	3.30E-06	ENSG00000164853
COL2A1	7.94	5.96	7.54E-09	3.35E-06	ENSG00000139219
USP6	5.91	5.35	7.82E-09	3.43E-06	ENSG00000129204
CABS1	7.96	-0.21	8.43E-09	3.64E-06	ENSG00000145309
AC019197.1	7.19	0.03	8.81E-09	3.74E-06	ENSG00000236283
RBMY2QP	7.58	-0.11	8.93E-09	3.74E-06	ENSG00000236718
MAB21L1	6.26	2.03	1.09E-08	4.40E-06	ENSG00000180660
KLHL14	6.81	1.43	1.09E-08	4.40E-06	ENSG00000197705
ERC2	6.08	1.81	1.10E-08	4.40E-06	ENSG00000187672
SHISAL2B	7.93	-0.30	1.15E-08	4.55E-06	ENSG00000145642
GPC5	6.65	1.33	1.35E-08	5.25E-06	ENSG00000179399

SYT10	7.84	-0.32	1.63E-08	6.27E-06	ENSG00000110975
BPIFB4	6.55	0.20	1.66E-08	6.28E-06	ENSG00000186191
CHRND	7.34	0.14	1.96E-08	7.32E-06	ENSG00000135902
YBX2	6.33	1.26	2.26E-08	8.35E-06	ENSG00000006047
KIF5A	4.79	2.21	2.35E-08	8.57E-06	ENSG00000155980
MYO16	6.13	2.03	2.40E-08	8.64E-06	ENSG00000041515
SLC15A1	7.24	0.10	3.34E-08	1.17E-05	ENSG00000088386
AGXT2	7.31	-0.28	3.35E-08	1.17E-05	ENSG00000113492
MKX	5.37	3.44	3.44E-08	1.19E-05	ENSG00000150051
TDRD12	6.15	4.59	3.55E-08	1.22E-05	ENSG00000173809
CACNA1B	5.25	1.75	3.98E-08	1.34E-05	ENSG00000148408
CCNB3	4.64	3.37	4.20E-08	1.40E-05	ENSG00000147082
ELAVL4	5.61	1.37	4.26E-08	1.41E-05	ENSG00000162374
SERHL2	4.86	2.80	4.32E-08	1.41E-05	ENSG00000183569
ALX4	5.71	0.79	5.29E-08	1.70E-05	ENSG00000052850
FRRS1L	6.26	0.59	5.37E-08	1.70E-05	ENSG00000260230
SLCO1A2	6.46	2.94	5.39E-08	1.70E-05	ENSG00000084453
DAZ1	7.62	-0.49	5.89E-08	1.82E-05	ENSG00000188120
DPY19L2P4	7.20	-0.36	5.89E-08	1.82E-05	ENSG00000235436
PIK3C2G	9.35	6.03	8.21E-08	2.50E-05	ENSG00000139144
TNFRSF19	3.74	5.12	8.49E-08	2.56E-05	ENSG00000127863
OTX2	7.55	-0.60	8.76E-08	2.61E-05	ENSG00000165588
NELL1	7.12	-0.41	9.66E-08	2.85E-05	ENSG00000165973
PHF21B	6.99	-0.10	1.05E-07	3.07E-05	ENSG00000056487
FGFR3	4.84	3.14	1.12E-07	3.23E-05	ENSG00000068078
VWDE	6.80	4.73	1.13E-07	3.23E-05	ENSG00000146530
ZNF793	4.74	2.54	1.16E-07	3.27E-05	ENSG00000188227
SPOCK3	7.56	1.65	1.28E-07	3.57E-05	ENSG00000196104
OR6C70	7.43	-0.62	1.29E-07	3.57E-05	ENSG00000184954
NDST4	6.20	1.58	1.30E-07	3.57E-05	ENSG00000138653
NPTX2	6.61	2.97	1.36E-07	3.71E-05	ENSG00000106236
MAPK4	6.32	0.24	1.43E-07	3.87E-05	ENSG00000141639
UNC5D	6.78	-0.12	1.60E-07	4.24E-05	ENSG00000156687
LGR5	6.76	5.98	1.60E-07	4.24E-05	ENSG00000139292
KCTD4	7.39	-0.65	1.76E-07	4.60E-05	ENSG00000180332
SNCAIP	3.65	5.56	1.94E-07	4.99E-05	ENSG00000064692
SOHLH1	6.95	-0.50	1.94E-07	4.99E-05	ENSG00000165643
BRINP1	6.67	0.70	2.33E-07	5.95E-05	ENSG00000078725
FRMPD1	6.83	0.95	2.44E-07	6.16E-05	ENSG00000070601

NKAIN4	6.06	1.12	2.49E-07	6.23E-05	ENSG00000101198
GAGE2A	7.29	1.23	2.75E-07	6.83E-05	ENSG00000189064
LINC01597	6.78	-0.28	3.05E-07	7.51E-05	ENSG00000205611
PAX1	7.26	-0.76	3.13E-07	7.63E-05	ENSG00000125813
MYLPF	7.22	-0.80	3.57E-07	8.63E-05	ENSG00000180209
PPP1R12BP1	7.21	-0.80	3.73E-07	8.89E-05	ENSG00000229238
ALX3	7.21	-0.80	3.74E-07	8.89E-05	ENSG00000156150
EMILIN3	5.91	1.94	3.78E-07	8.92E-05	ENSG00000183798
FAM3B	5.15	0.78	4.37E-07	1.02E-04	ENSG00000183844
NKD1	4.57	2.99	4.40E-07	1.02E-04	ENSG00000140807
CXorf67	6.16	0.07	4.44E-07	1.02E-04	ENSG00000187690
BMP5	7.41	4.55	5.32E-07	1.21E-04	ENSG00000112175
BEND4	6.17	-0.10	5.36E-07	1.21E-04	ENSG00000188848
PAX2	7.15	-0.84	5.48E-07	1.23E-04	ENSG00000075891
LEMD1	7.11	-0.88	6.29E-07	1.40E-04	ENSG00000186007
ANKRD18A	5.12	2.96	6.37E-07	1.41E-04	ENSG00000180071
CCDC178	6.53	1.04	6.48E-07	1.41E-04	ENSG00000166960
COL9A2	3.49	4.20	6.50E-07	1.41E-04	ENSG00000049089
DDX11L10	7.05	-0.11	6.69E-07	1.44E-04	ENSG00000233614
COL13A1	4.18	4.66	6.81E-07	1.46E-04	ENSG00000197467
VSTM2A	6.71	-0.74	7.08E-07	1.50E-04	ENSG00000170419
AC013470.1	5.80	-0.52	7.11E-07	1.50E-04	ENSG00000224667
UBE3AP2	5.24	1.06	7.52E-07	1.57E-04	ENSG00000228941
OPRK1	7.03	-0.92	8.08E-07	1.67E-04	ENSG00000082556
RPL23AP96	7.03	-0.92	8.09E-07	1.67E-04	ENSG00000250752
FSTL5	6.64	3.06	8.93E-07	1.83E-04	ENSG00000168843
DKK2	4.90	2.40	9.23E-07	1.87E-04	ENSG00000155011
LMX1A	6.30	-0.68	9.24E-07	1.87E-04	ENSG00000162761
HEPHL1	6.37	2.13	9.61E-07	1.91E-04	ENSG00000181333
ARMC4	4.52	5.56	9.62E-07	1.91E-04	ENSG00000169126
RTL1	6.99	-0.96	1.11E-06	2.19E-04	ENSG00000254656
TG	3.29	5.75	1.12E-06	2.19E-04	ENSG00000042832
TNNI1	6.86	-0.60	1.29E-06	2.50E-04	ENSG00000159173
IRX1	7.02	0.19	1.31E-06	2.53E-04	ENSG00000170549
NELL2	4.17	4.92	1.39E-06	2.67E-04	ENSG00000184613
PRDM11	3.17	4.89	1.40E-06	2.67E-04	ENSG00000019485
DCHS2	5.56	2.56	1.43E-06	2.70E-04	ENSG00000197410
DLX4	4.86	0.28	1.50E-06	2.81E-04	ENSG00000108813
CSN3	6.03	-0.45	1.50E-06	2.81E-04	ENSG00000171209

ATXN7L2	3.60	3.67	1.58E-06	2.94E-04	ENSG00000162650
NTS	6.51	2.20	1.65E-06	3.04E-04	ENSG00000133636
AC068446.3	6.45	-0.54	1.79E-06	3.27E-04	ENSG00000258883
BCL11A	4.85	2.47	1.80E-06	3.27E-04	ENSG00000119866
IRS4	5.65	0.51	1.87E-06	3.38E-04	ENSG00000133124
C2CD4C	6.41	-0.57	2.04E-06	3.67E-04	ENSG00000183186
IL17RD	3.37	4.03	2.25E-06	4.02E-04	ENSG00000144730
RNF175	5.51	1.90	2.30E-06	4.08E-04	ENSG00000145428
ANKRD33	5.59	1.03	2.39E-06	4.22E-04	ENSG00000167612
GRIA1	6.21	3.49	2.42E-06	4.24E-04	ENSG00000155511
SELENOV	6.27	-0.47	2.63E-06	4.57E-04	ENSG00000186838
KIRREL2	6.06	-0.82	2.66E-06	4.61E-04	ENSG00000126259
HMGCLL1	6.69	-0.70	2.72E-06	4.69E-04	ENSG00000146151
PRKG2	5.29	0.68	2.78E-06	4.76E-04	ENSG00000138669
AGMO	5.30	4.86	2.89E-06	4.91E-04	ENSG00000187546
RPL39P29	6.73	-0.33	3.13E-06	5.30E-04	ENSG00000236860
FGF14	6.44	1.18	3.73E-06	6.27E-04	ENSG00000102466
PTCHD4	4.11	3.45	3.76E-06	6.27E-04	ENSG00000244694
ZNF571-AS1	5.31	0.42	3.83E-06	6.34E-04	ENSG00000267470
ERBB4	5.95	4.28	3.84E-06	6.34E-04	ENSG00000178568
IGLV11-55	5.51	0.35	4.04E-06	6.61E-04	ENSG00000211641
NCAN	5.96	-0.65	4.05E-06	6.61E-04	ENSG00000130287
SORCS1	6.32	0.65	4.15E-06	6.73E-04	ENSG00000108018
PEX5L	5.93	-0.47	4.27E-06	6.87E-04	ENSG00000114757
RORB	6.47	0.63	4.50E-06	7.21E-04	ENSG00000198963
MAG	5.80	0.82	4.59E-06	7.32E-04	ENSG00000105695
INTS4P2	5.46	-0.32	5.10E-06	8.08E-04	ENSG00000273024
UGT3A2	6.27	-1.07	5.32E-06	8.38E-04	ENSG00000168671
AC008805.2	5.98	-0.19	5.51E-06	8.64E-04	ENSG00000267555
CPA6	5.23	-0.50	5.61E-06	8.74E-04	ENSG00000165078
NOS1	5.92	-0.95	5.67E-06	8.78E-04	ENSG00000089250
CNTNAP5	5.28	0.24	5.74E-06	8.84E-04	ENSG00000155052
PROM1	5.28	3.41	5.87E-06	8.99E-04	ENSG00000007062
MYOG	6.63	-1.19	6.03E-06	9.18E-04	ENSG00000122180
DRD1	6.62	-1.19	6.11E-06	9.21E-04	ENSG00000184845
AC109466.1	6.62	-1.19	6.11E-06	9.21E-04	ENSG00000241956
CCDC169	4.94	0.81	6.23E-06	9.35E-04	ENSG00000242715
AC112206.4	5.79	-0.52	6.60E-06	9.84E-04	ENSG00000250669
OGDHL	5.82	-0.21	6.67E-06	9.89E-04	ENSG00000197444

OR8G5	6.19	-1.12	6.81E-06	1.00E-03	ENSG00000255298
TAC1	6.19	-1.12	6.82E-06	1.00E-03	ENSG00000006128
PHF2P2	6.42	3.93	7.18E-06	1.05E-03	ENSG00000226057
GABRB1	6.05	-0.33	7.48E-06	1.09E-03	ENSG00000163288
NBEA	3.32	7.71	7.73E-06	1.12E-03	ENSG00000172915
TSHR	4.51	1.41	7.76E-06	1.12E-03	ENSG00000165409
HNRNPA1P2	4.14	2.72	7.99E-06	1.14E-03	ENSG00000237285
FAM9A	6.54	-1.24	8.00E-06	1.14E-03	ENSG00000183304
KCNA2	4.96	2.29	8.05E-06	1.14E-03	ENSG00000177301
ESPNP	5.57	-0.13	8.44E-06	1.19E-03	ENSG00000268869
MPL	5.29	1.10	8.67E-06	1.22E-03	ENSG00000117400
AC097478.1	6.49	-0.51	8.72E-06	1.22E-03	ENSG00000251095
ATRNL1	4.61	4.22	8.80E-06	1.22E-03	ENSG00000107518
SRD5A2	6.09	-0.83	9.67E-06	1.34E-03	ENSG00000277893
SPHKAP	5.99	0.09	1.01E-05	1.39E-03	ENSG00000153820
KRTAP11-1	6.51	-1.30	1.11E-05	1.52E-03	ENSG00000182591
RXFP2	5.54	-0.96	1.12E-05	1.52E-03	ENSG00000133105
LHFPL1	5.99	-0.86	1.14E-05	1.55E-03	ENSG00000182508
ADCY5	4.24	3.18	1.22E-05	1.65E-03	ENSG00000173175
AL391261.2	6.46	-1.29	1.28E-05	1.72E-03	ENSG00000258847
SULT1C2	5.59	0.95	1.30E-05	1.73E-03	ENSG00000198203
GPR50	5.49	-1.01	1.36E-05	1.81E-03	ENSG00000102195
LRFN2	5.44	-1.00	1.49E-05	1.98E-03	ENSG00000156564
TGFA	4.16	0.63	1.54E-05	2.02E-03	ENSG00000163235
RGS20	5.07	1.27	1.54E-05	2.02E-03	ENSG00000147509
MC4R	5.80	-0.85	1.55E-05	2.02E-03	ENSG00000166603
PKHD1	4.68	2.72	1.56E-05	2.02E-03	ENSG00000170927
PTPRVP	5.07	-0.44	1.59E-05	2.05E-03	ENSG00000243323
RTP3	6.36	-1.34	1.68E-05	2.17E-03	ENSG00000163825
WDR72	6.03	0.10	1.74E-05	2.23E-03	ENSG00000166415
SLC1A3	2.62	5.81	1.77E-05	2.26E-03	ENSG00000079215
TRPC5	6.34	-1.34	1.82E-05	2.30E-03	ENSG00000072315
ODAM	5.76	2.31	1.82E-05	2.30E-03	ENSG00000109205
MRPL9P1	5.94	-1.20	1.89E-05	2.38E-03	ENSG00000254305
DAB1	5.45	0.81	1.92E-05	2.39E-03	ENSG00000173406
PRSS44	4.71	-0.02	1.93E-05	2.39E-03	ENSG00000226074
KCNA4	5.91	-0.95	1.93E-05	2.39E-03	ENSG00000182255
ZNF157	4.34	0.17	1.98E-05	2.44E-03	ENSG00000147117
TGS1	2.41	6.84	2.01E-05	2.48E-03	ENSG00000137574

KNDC1	5.54	1.72	2.06E-05	2.52E-03	ENSG00000171798
BOC	3.44	5.86	2.17E-05	2.64E-03	ENSG00000144857
CCDC162P	4.05	2.98	2.19E-05	2.66E-03	ENSG00000203799
MPPED2	4.81	3.36	2.21E-05	2.67E-03	ENSG00000066382
EBF3	3.00	5.06	2.22E-05	2.68E-03	ENSG00000108001
PSPC1P1	4.51	-0.30	2.26E-05	2.71E-03	ENSG00000227879
ASNSP1	5.41	0.64	2.33E-05	2.78E-03	ENSG00000248498
TTPA	5.83	-0.19	2.51E-05	2.97E-03	ENSG00000137561
WFIKKN2	5.04	-0.76	2.51E-05	2.97E-03	ENSG00000173714
ASXL3	5.23	4.34	2.54E-05	2.99E-03	ENSG00000141431
ROBO2	5.32	4.61	2.59E-05	3.03E-03	ENSG00000185008
RPL21P71	5.55	-0.91	2.59E-05	3.03E-03	ENSG00000239412
PAPPA2	4.93	4.02	2.80E-05	3.25E-03	ENSG00000116183
KC6	5.65	-0.93	2.85E-05	3.30E-03	ENSG00000267313
SLCO4C1	4.48	1.01	2.92E-05	3.37E-03	ENSG00000173930
11-Mar	6.83	1.10	2.96E-05	3.40E-03	ENSG00000183654
SYN3	5.18	1.52	3.20E-05	3.65E-03	ENSG00000185666
FAM135B	4.93	-0.61	3.21E-05	3.65E-03	ENSG00000147724
CYP3A4	5.11	-0.65	3.22E-05	3.65E-03	ENSG00000160868
LINC01529	5.84	-1.25	3.24E-05	3.66E-03	ENSG00000225872
SLC9A2	4.65	-0.41	3.25E-05	3.66E-03	ENSG00000115616
AACSP1	5.29	1.12	3.30E-05	3.70E-03	ENSG00000250420
NPHS1	5.28	-0.43	3.32E-05	3.71E-03	ENSG00000161270
PCDH15	5.55	2.52	3.38E-05	3.76E-03	ENSG00000150275
OPTN	-4.54	8.09	3.41E-05	3.78E-03	ENSG00000123240
AC004540.2	5.71	-0.79	3.44E-05	3.79E-03	ENSG00000225792
SMYD1	6.09	-1.10	3.55E-05	3.89E-03	ENSG00000115593
SHANK2	6.09	3.61	3.55E-05	3.89E-03	ENSG00000162105
GRIN2C	4.33	0.70	3.81E-05	4.13E-03	ENSG00000161509
NNMT	-5.05	5.34	3.81E-05	4.13E-03	ENSG00000166741
AL357373.1	5.00	-1.05	3.81E-05	4.13E-03	ENSG00000233216
AL603840.1	5.86	-0.13	3.95E-05	4.26E-03	ENSG00000234810
ZFHX4	4.05	8.66	3.97E-05	4.27E-03	ENSG00000091656
ZNF236	2.32	6.99	3.99E-05	4.27E-03	ENSG00000130856
MYB	4.25	3.26	4.09E-05	4.37E-03	ENSG00000118513
ZNF232	2.97	5.06	4.27E-05	4.53E-03	ENSG00000167840
GABRG3	6.14	-1.45	4.35E-05	4.60E-03	ENSG00000182256
CD101	3.51	3.71	4.44E-05	4.68E-03	ENSG00000134256
SH3GL3	4.15	2.20	4.78E-05	5.02E-03	ENSG00000140600

SYT9	4.73	-0.35	4.88E-05	5.10E-03	ENSG00000170743
NMUR2	5.70	-1.36	4.98E-05	5.19E-03	ENSG00000132911
CPAMD8	4.00	3.47	5.01E-05	5.21E-03	ENSG00000160111
ZNF516	3.22	5.88	5.36E-05	5.55E-03	ENSG00000101493
GDF7	4.35	-0.01	5.58E-05	5.76E-03	ENSG00000143869
MIR548F2	6.00	-1.15	5.65E-05	5.80E-03	ENSG00000221782
B3GALT1	5.61	-0.66	5.68E-05	5.80E-03	ENSG00000172318
PTPN20	4.15	3.20	5.70E-05	5.80E-03	ENSG00000204179
SPIDR	2.18	7.81	5.71E-05	5.80E-03	ENSG00000164808
AC104057.1	6.04	-1.51	5.79E-05	5.86E-03	ENSG00000230386
GAGE1	6.04	-1.51	5.89E-05	5.94E-03	ENSG00000205777
SLAIN1	4.09	3.18	5.94E-05	5.97E-03	ENSG00000139737
LCT	3.94	2.23	6.13E-05	6.13E-03	ENSG00000115850
ATP8A2	3.74	2.91	6.18E-05	6.17E-03	ENSG00000132932
MYO5B	3.72	4.20	6.41E-05	6.37E-03	ENSG00000167306
TSBP1-AS1	4.49	0.95	6.63E-05	6.56E-03	ENSG00000225914
COL20A1	4.40	-0.28	6.65E-05	6.56E-03	ENSG00000101203
LGALS3BP	-5.26	5.99	6.84E-05	6.73E-03	ENSG00000108679
KIRREL3	4.99	-0.89	7.05E-05	6.91E-03	ENSG00000149571
PSMC1P9	5.14	-0.64	7.16E-05	6.99E-03	ENSG00000255993
SPRY2	2.47	5.86	7.53E-05	7.33E-03	ENSG00000136158
COL24A1	3.30	6.09	7.56E-05	7.34E-03	ENSG00000171502
FREM2	5.53	2.42	7.73E-05	7.45E-03	ENSG00000150893
LRRN3	4.54	2.33	7.75E-05	7.45E-03	ENSG00000173114
CTNNA2	6.40	0.41	7.77E-05	7.45E-03	ENSG00000066032
FAT2	4.14	2.60	7.81E-05	7.45E-03	ENSG00000086570
AC008750.2	4.34	-0.78	7.82E-05	7.45E-03	ENSG00000255441
ALB	4.17	1.10	7.84E-05	7.45E-03	ENSG00000163631
NKAIN2	5.00	-0.19	7.91E-05	7.47E-03	ENSG00000188580
LRRIQ1	4.17	4.28	7.91E-05	7.47E-03	ENSG00000133640
AC079601.1	3.74	0.99	7.94E-05	7.47E-03	ENSG00000257225
THSD7A	3.45	6.39	8.03E-05	7.53E-03	ENSG00000005108
BX649567.1	4.86	-0.88	8.17E-05	7.64E-03	ENSG00000284737
BRIP1	2.28	6.68	8.64E-05	8.05E-03	ENSG00000136492
IGKV2OR22-3	5.90	-1.56	8.71E-05	8.09E-03	ENSG00000253460
FAM19A1	5.59	-1.41	8.96E-05	8.29E-03	ENSG00000183662
OSBP2	3.33	3.41	8.99E-05	8.29E-03	ENSG00000184792
MCM4	2.38	6.91	9.29E-05	8.54E-03	ENSG00000104738
CYP24A1	5.74	1.26	9.31E-05	8.54E-03	ENSG00000019186

MAGEB1	6.00	0.90	9.40E-05	8.59E-03	ENSG00000214107
CD22	4.30	3.37	9.55E-05	8.70E-03	ENSG00000012124
AC004980.2	5.49	-1.16	9.59E-05	8.70E-03	ENSG00000214243
CHGB	4.52	-0.17	1.01E-04	9.13E-03	ENSG00000089199
PAX9	5.59	1.98	1.04E-04	9.38E-03	ENSG00000198807
GAREM2	3.78	2.20	1.05E-04	9.42E-03	ENSG00000157833
PEG3	4.84	2.53	1.06E-04	9.54E-03	ENSG00000198300
RIMBP2	5.42	0.17	1.08E-04	9.63E-03	ENSG00000060709
FAM169A	3.67	1.86	1.17E-04	1.04E-02	ENSG00000198780
AC011447.4	4.09	0.65	1.18E-04	1.05E-02	ENSG00000267771
OR13C9	5.44	-1.46	1.29E-04	1.14E-02	ENSG00000136839
PRSS12	4.11	5.91	1.30E-04	1.14E-02	ENSG00000164099
KCNK2	5.45	2.02	1.32E-04	1.16E-02	ENSG00000082482
SLC6A15	5.72	1.68	1.34E-04	1.17E-02	ENSG00000072041
PTPRQ	3.90	2.90	1.34E-04	1.17E-02	ENSG00000139304
ARPP21	4.39	-0.32	1.36E-04	1.18E-02	ENSG00000172995
SLITRK1	5.15	0.81	1.36E-04	1.18E-02	ENSG00000178235
MED15P4	5.64	0.73	1.38E-04	1.20E-02	ENSG00000224679
ASTN2	2.89	3.79	1.43E-04	1.23E-02	ENSG00000148219
AC135068.11	5.77	-0.57	1.44E-04	1.24E-02	ENSG00000285491
SDR9C7	3.93	-0.10	1.50E-04	1.29E-02	ENSG00000170426
MIER3	2.31	5.83	1.51E-04	1.30E-02	ENSG00000155545
CXCL14	5.87	1.13	1.54E-04	1.31E-02	ENSG00000145824
RPL29P3	5.80	-1.63	1.56E-04	1.33E-02	ENSG00000243730
SSX7	5.15	-1.37	1.58E-04	1.33E-02	ENSG00000187754
SACS	2.16	9.29	1.58E-04	1.33E-02	ENSG00000151835
USH2A	3.80	0.11	1.60E-04	1.35E-02	ENSG00000042781
TUBAL3	5.78	-1.63	1.61E-04	1.35E-02	ENSG00000178462
PRKDC	2.15	10.22	1.62E-04	1.36E-02	ENSG00000253729
ITPRID1	6.21	0.47	1.62E-04	1.36E-02	ENSG00000180347
MARCOL	5.78	-1.63	1.64E-04	1.36E-02	ENSG00000248109
BX284668.3	5.78	-1.63	1.64E-04	1.36E-02	ENSG00000230239
EDN3	5.88	3.51	1.65E-04	1.36E-02	ENSG00000124205
TMTC2	3.41	5.81	1.66E-04	1.37E-02	ENSG00000179104
POU3F3	4.44	-1.08	1.67E-04	1.37E-02	ENSG00000198914
TPO	5.22	-0.57	1.71E-04	1.40E-02	ENSG00000115705
PCOLCE-AS1	3.38	2.07	1.73E-04	1.41E-02	ENSG00000224729
ETNPPL	5.76	-1.63	1.73E-04	1.41E-02	ENSG00000164089
COL9A3	4.03	3.88	1.75E-04	1.42E-02	ENSG00000092758

ADGRG2	4.80	2.53	1.90E-04	1.54E-02	ENSG00000173698
BRSK2	4.67	0.32	1.94E-04	1.57E-02	ENSG00000174672
VGLL2	5.64	-1.35	1.95E-04	1.57E-02	ENSG00000170162
HOOK1	3.54	2.20	1.98E-04	1.59E-02	ENSG00000134709
PAK5	5.69	-1.71	1.98E-04	1.59E-02	ENSG00000101349
PRSS42	3.72	1.00	2.01E-04	1.61E-02	ENSG00000178055
GRIA4	4.61	1.08	2.07E-04	1.65E-02	ENSG00000152578
LGSN	5.04	0.00	2.10E-04	1.67E-02	ENSG00000146166
NMU	3.88	-0.48	2.16E-04	1.71E-02	ENSG00000109255
SLC7A3	6.18	0.59	2.17E-04	1.71E-02	ENSG00000165349
EVX1	5.64	-1.70	2.25E-04	1.77E-02	ENSG00000106038
ANKRD30BP2	5.64	-1.70	2.25E-04	1.77E-02	ENSG00000224309
EIF4HP2	3.56	0.39	2.26E-04	1.77E-02	ENSG00000237977
BMS1P7	4.96	-1.21	2.28E-04	1.78E-02	ENSG00000270025
FKBP4P1	4.76	-1.19	2.34E-04	1.82E-02	ENSG00000251463
AL513318.2	5.63	-1.70	2.34E-04	1.82E-02	ENSG00000269994
MIR181B2	3.79	-0.55	2.35E-04	1.82E-02	ENSG00000207737
IL17RB	3.68	0.08	2.36E-04	1.82E-02	ENSG00000056736
SPEF2	2.72	5.00	2.37E-04	1.82E-02	ENSG00000152582
ADGRD2	5.63	-1.70	2.38E-04	1.82E-02	ENSG00000180264
SLC26A7	3.82	4.07	2.38E-04	1.82E-02	ENSG00000147606
SLC24A2	5.12	1.15	2.39E-04	1.82E-02	ENSG00000155886
OR8A1	5.20	-1.03	2.39E-04	1.82E-02	ENSG00000196119
OR4N3P	4.78	-1.20	2.41E-04	1.83E-02	ENSG00000259435
C16orf96	3.77	0.91	2.42E-04	1.83E-02	ENSG00000205832
GCGR	4.96	-1.21	2.44E-04	1.84E-02	ENSG00000215644
GRK1	4.41	-0.85	2.45E-04	1.85E-02	ENSG00000185974
AC073109.1	4.07	2.36	2.47E-04	1.86E-02	ENSG00000235920
RPL7L1P11	4.45	-0.90	2.52E-04	1.89E-02	ENSG00000176970
FGFR2	3.37	4.50	2.78E-04	2.08E-02	ENSG00000066468
CNTNAP3	3.19	3.56	2.88E-04	2.15E-02	ENSG00000106714
SVIL2P	3.15	2.75	2.99E-04	2.22E-02	ENSG00000234814
CASQ2	4.66	1.57	3.00E-04	2.23E-02	ENSG00000118729
RPS26P15	4.34	1.21	3.02E-04	2.23E-02	ENSG00000223416
PIANP	4.62	-0.16	3.03E-04	2.23E-02	ENSG00000139200
TSPAN7	3.81	0.85	3.03E-04	2.23E-02	ENSG00000156298
PDE1C	2.98	5.77	3.05E-04	2.24E-02	ENSG00000154678
AC027682.3	3.35	1.36	3.07E-04	2.25E-02	ENSG00000260894
GRM1	4.07	2.38	3.09E-04	2.26E-02	ENSG00000152822

NSUN7	3.13	1.96	3.10E-04	2.26E-02	ENSG00000179299
F7	4.70	0.16	3.12E-04	2.27E-02	ENSG00000057593
NOTUM	4.75	0.34	3.14E-04	2.28E-02	ENSG00000185269
CNTFR	3.57	2.87	3.15E-04	2.28E-02	ENSG00000122756
WNK4	4.52	3.63	3.25E-04	2.35E-02	ENSG00000126562
HS3ST5	4.63	-0.52	3.29E-04	2.37E-02	ENSG00000249853
STK26	2.82	3.57	3.33E-04	2.39E-02	ENSG00000134602
CNKSR2	4.53	2.60	3.35E-04	2.40E-02	ENSG00000149970
FAM183A	3.89	0.69	3.40E-04	2.43E-02	ENSG00000186973
GNGT1	4.72	-1.07	3.45E-04	2.46E-02	ENSG00000127928
RIC3	3.90	1.66	3.66E-04	2.60E-02	ENSG00000166405
KCNT1	4.47	-0.80	3.67E-04	2.60E-02	ENSG00000107147
NRK	4.43	4.33	3.69E-04	2.60E-02	ENSG00000123572
IL11RA	2.56	5.33	3.70E-04	2.61E-02	ENSG00000137070
AL021920.2	4.96	0.94	3.74E-04	2.63E-02	ENSG00000280114
OR8G2P	5.11	-0.82	3.79E-04	2.66E-02	ENSG00000181214
SP8	5.16	-1.58	3.81E-04	2.66E-02	ENSG00000164651
WNT16	5.16	-1.58	3.81E-04	2.66E-02	ENSG00000002745
HOXA13	3.91	2.18	3.86E-04	2.68E-02	ENSG00000106031
PLPPR5	4.57	-1.10	3.95E-04	2.74E-02	ENSG00000117598
FIGNL1	2.57	5.00	3.96E-04	2.74E-02	ENSG00000132436
IGDCC3	3.85	0.91	3.97E-04	2.74E-02	ENSG00000174498
DDX3P3	4.84	-1.08	4.00E-04	2.75E-02	ENSG00000251571
MUM1L1	5.40	1.31	4.31E-04	2.96E-02	ENSG00000157502
GABRB3	3.96	0.21	4.35E-04	2.98E-02	ENSG00000166206
AC025569.1	4.23	2.87	4.35E-04	2.98E-02	ENSG00000258168
LRRC66	2.93	3.17	4.39E-04	3.00E-02	ENSG00000188993
IGKV1OR22-1	4.82	-0.97	4.40E-04	3.00E-02	ENSG00000253481
ONECUT2	5.28	-0.14	4.43E-04	3.00E-02	ENSG00000119547
WNK2	3.61	3.13	4.44E-04	3.00E-02	ENSG00000165238
XDH	4.72	3.22	4.44E-04	3.00E-02	ENSG00000158125
RADX	3.55	3.20	4.50E-04	3.03E-02	ENSG00000147231
TDRD5	3.90	-0.12	4.52E-04	3.04E-02	ENSG00000162782
GABRG2	6.11	2.17	4.67E-04	3.12E-02	ENSG00000113327
TPTE2P1	4.16	1.00	4.67E-04	3.12E-02	ENSG00000253771
CATSPER4	4.78	-1.32	4.74E-04	3.16E-02	ENSG00000188782
PCDH11X	5.12	3.57	4.76E-04	3.16E-02	ENSG00000102290
ZNF711	3.53	6.18	4.76E-04	3.16E-02	ENSG00000147180
NBEAP1	4.33	1.29	4.80E-04	3.18E-02	ENSG00000258590

REG1A	5.52	-1.78	4.84E-04	3.20E-02	ENSG00000115386
TPTE2P2	5.52	-1.78	4.84E-04	3.20E-02	ENSG00000272281
ANKRD20A3	5.07	-1.35	4.87E-04	3.21E-02	ENSG00000276203
OR2M3	5.06	-1.65	4.99E-04	3.28E-02	ENSG00000228198
IL20RA	5.18	0.46	5.07E-04	3.32E-02	ENSG00000016402
CENPIP1	5.69	-0.25	5.15E-04	3.36E-02	ENSG00000224778
AL512324.3	5.11	-1.18	5.16E-04	3.36E-02	ENSG00000278531
PTH1R	2.70	4.86	5.16E-04	3.36E-02	ENSG00000160801
LINC01694	5.02	-1.65	5.24E-04	3.40E-02	ENSG00000233922
CACNA2D2	3.60	2.42	5.27E-04	3.41E-02	ENSG00000007402
GOLGA8S	4.49	-1.28	5.27E-04	3.41E-02	ENSG00000261739
ZCCHC12	3.67	-0.09	5.31E-04	3.42E-02	ENSG00000174460
MYEOV	4.00	-0.19	5.34E-04	3.43E-02	ENSG00000172927
MT2A	-5.14	5.87	5.37E-04	3.44E-02	ENSG00000125148
PDZRN4	5.08	0.43	5.46E-04	3.50E-02	ENSG00000165966
VWC2	5.46	-1.76	5.50E-04	3.51E-02	ENSG00000188730
RBM24	4.13	0.96	5.53E-04	3.53E-02	ENSG00000112183
SNRPCP5	5.46	-1.76	5.57E-04	3.54E-02	ENSG00000255522
ASIC2	5.46	-1.76	5.58E-04	3.54E-02	ENSG00000108684
SGCZ	5.33	-1.53	5.59E-04	3.54E-02	ENSG00000185053
NBPF13P	3.97	0.41	5.62E-04	3.55E-02	ENSG00000227242
FLJ16779	5.00	-1.64	5.67E-04	3.57E-02	ENSG00000275620
DLX6-AS1	4.91	-1.04	5.79E-04	3.64E-02	ENSG00000231764
LRRC9	5.44	-1.76	5.87E-04	3.68E-02	ENSG00000131951
GABRB2	4.43	0.01	5.89E-04	3.68E-02	ENSG00000145864
TAB3-AS2	4.37	-1.26	5.95E-04	3.72E-02	ENSG00000235512
TMEM272	4.32	-1.25	5.98E-04	3.73E-02	ENSG00000281106
CNTNAP3C	4.77	-1.54	6.01E-04	3.73E-02	ENSG00000283378
UNC79	3.68	1.21	6.07E-04	3.76E-02	ENSG00000133958
SEMA3E	5.13	3.25	6.17E-04	3.82E-02	ENSG00000170381
GRPR	3.47	0.72	6.20E-04	3.83E-02	ENSG00000126010
AC073316.1	5.05	1.96	6.38E-04	3.93E-02	ENSG00000217455
ANKRD20A8P	4.72	6.24	6.42E-04	3.94E-02	ENSG00000229089
THBS4	3.60	6.21	6.42E-04	3.94E-02	ENSG00000113296
OR10D3	5.40	-0.68	6.56E-04	4.02E-02	ENSG00000197309
PIWIL2	3.35	3.46	6.62E-04	4.04E-02	ENSG00000197181
MVP	-3.86	6.84	6.73E-04	4.10E-02	ENSG00000013364
ADGRV1	4.05	5.60	6.84E-04	4.16E-02	ENSG00000164199
ZSCAN23	3.35	2.56	6.91E-04	4.20E-02	ENSG00000187987

IFITM2	-4.15	6.30	7.04E-04	4.26E-02	ENSG00000185201
PLAG1	3.51	4.61	7.11E-04	4.30E-02	ENSG00000181690
SLC25A21	5.53	-0.57	7.13E-04	4.30E-02	ENSG00000183032
CNBD1	3.98	-0.51	7.24E-04	4.36E-02	ENSG00000176571
SMPD4P1	4.41	-1.20	7.26E-04	4.36E-02	ENSG00000223553
AC007608.3	4.23	-1.31	7.35E-04	4.40E-02	ENSG00000260249
SIM1	4.86	2.68	7.36E-04	4.40E-02	ENSG00000112246
CCKAR	4.31	-0.54	7.41E-04	4.42E-02	ENSG00000163394
MAP3K8	-3.56	5.44	7.46E-04	4.44E-02	ENSG00000107968
PNMA3	3.81	2.77	7.52E-04	4.47E-02	ENSG00000183837
SYT2	4.51	-0.67	7.63E-04	4.53E-02	ENSG00000143858
ADH5P3	5.33	-1.85	7.74E-04	4.58E-02	ENSG00000223694
ARG1	4.47	-0.57	7.82E-04	4.62E-02	ENSG00000118520
SLC1A2	3.35	2.08	8.01E-04	4.72E-02	ENSG00000110436
MYO15A	3.27	4.68	8.07E-04	4.75E-02	ENSG00000091536
CYTL1	3.50	1.83	8.13E-04	4.77E-02	ENSG00000170891
BHLHE40	-3.40	7.29	8.27E-04	4.84E-02	ENSG00000134107
ATAD5	1.99	6.06	8.42E-04	4.92E-02	ENSG00000176208
PNMT	4.59	-1.60	8.45E-04	4.93E-02	ENSG00000141744
FAM124A	3.03	1.56	8.55E-04	4.97E-02	ENSG00000150510
